# An Unusual Case of Psoriatic Arthritis With Secondary Lingual Lesions, Resembling Geographic Tongue

**DOI:** 10.7759/cureus.63439

**Published:** 2024-06-29

**Authors:** Vasileios Zisis, Athina Theodoridou, Eleftherios Anagnostou, Athanasios Poulopoulos, Dimitrios Andreadis

**Affiliations:** 1 Oral Medicine/Pathology, Aristotle University of Thessaloniki, Thessaloniki, GRC; 2 Rheumatology, Aristotle University of Thessaloniki, Thessaloniki, GRC

**Keywords:** geographic tongue, munro’s micro abscess, oral, psoriatic arthritis, psoriaris

## Abstract

Psoriatic arthritis (PsA) is a chronic inflammatory condition that impacts a significant proportion of individuals diagnosed with psoriasis. This report presents a rare case of a patient diagnosed with PsA who never had active psoriatic skin lesions but only a family history of psoriasis, with secondary lingual lesions, resembling geographic tongue (GT). A male patient, 24 years old, was referred with two painless erythematous areas resembling (without whitish borders, as in GT) rounded atrophic lesions on the dorsal surface of the tongue, resistant to any kind of antimicrobial/antifungal treatment for more than six months. The patient was diagnosed with PsA two years ago fulfilling the CASPAR (ClASsification for Psoriatic ARthritis) criteria. The patient never had active psoriatic skin lesions, but his father had psoriasis. The biopsy of lingual lesions showed moderate hyperkeratosis, spongiosis, and diffuse inflammatory infiltration of lymphocytes and neutrophils in the lamina propria as well as in the stratified squamous epithelium forming Munro’s microabscesses at the superficial layers. The manifestation of the atypical psoriasiform, GT-like lingual lesions was considered as part of psoriasis manifestations and the patient was advised to follow regular checkups so that any major exacerbation of the systematic symptoms could be preemptively avoided. Not only GT but also atypical lingual GT-like reddish oral lesions may be considered as transient forms of psoriasis supporting an early diagnosis and monitoring of psoriasis/PsA.

## Introduction

Psoriatic arthritis (PsA) is a chronic inflammatory condition that impacts a significant proportion, ranging from 14.0% to 22.7% of the individuals diagnosed with psoriasis [1-3]. The prevalence of PsA exhibits variation across different regions, with European psoriatic patients experiencing an incidence rate of 22.7%, South American patients at 21.5%, North American patients at 19.5%, African patients at 15.5%, and Asian patients at 14.0% [3]. A prevalence rate of 10.5% of PsA was noticed, among psoriatic patients who were initially seen at medical facilities [4]. The incidence of PsA exhibits a range of 0.19-0.25% [5,6]. The primary symptoms encompass peripheral arthritis, spondylitis, enthesitis, and/or dactylitis [7].

PsA is classified into five distinct subgroups: (1) asymmetric oligoarthritis, (2) significant distal interphalangeal joint involvement, (3) symmetric polyarthritis, (4) predominant axial involvement, and (5) arthritis mutilans [8]. It is characterized by significant heterogeneity, with varying clinical characteristics [9]. The clinical characteristics of this condition are not pathognomonic since they may be observed in other disorders, including reactive arthritis, osteoarthritis, and ankylosing spondylitis [10]. Therefore, these shared and intersecting characteristics pose difficulties in achieving a precise diagnosis of PsA [8]. Poor physical condition and disability issues may arise due to delayed diagnosis of PsA [11,12].

Early PsA (ePsA) refers to the presence of inflammatory joint symptoms and signs that are consistent with PsA and have been present for less than 24 months [13]. Typically, ePsA first manifests as enthesoarthritis, with a notable likelihood of progressing to erosive and deforming arthritis during the first year of the disease [14,15]. The presence of oral cavity lesions is infrequent [16-18]. Most probably, the absence of distinct symptoms such as discomfort or burning sensations, which are characteristic of psoriasis, may lead to a significant number of unreported cases. The epithelial turnover in individuals with psoriasis is expedited, resulting in a duration of three to seven days, as opposed to the typical timeframe of roughly 28 days [17]. This faster turnover poses challenges in effectively monitoring and detecting alterations in the oral cavity during such a short period. Patients may also fail to recognize oral manifestations due to experiencing significant distress from skin symptoms, which may divert their focus away from potential oral symptoms [19].

In the oral mucosa, alterations manifest as inflammation in the corners of the mouth, known as cheilitis, or as a condition called "perleche" characterized by erythema, dryness, and exfoliation [16]. Additionally, the vermilion of the lips may be affected, leading to recurring exfoliation and dryness. Occasionally, however rarely, the alterations manifest in the gingival region, presenting as exfoliation gingivitis with corresponding clinical features [16,18,20,21]. Lesions located in the lingual portion of the oral cavity are frequently observed in the form of geographic tongue or fissured tongue [20,22]. This report presents a rare case of ePsA with concurrent lingual psoriatic lesions, resembling geographic tongue (GT), without any pre-existing cutaneous lesions.

## Case presentation

**Figure 1:**
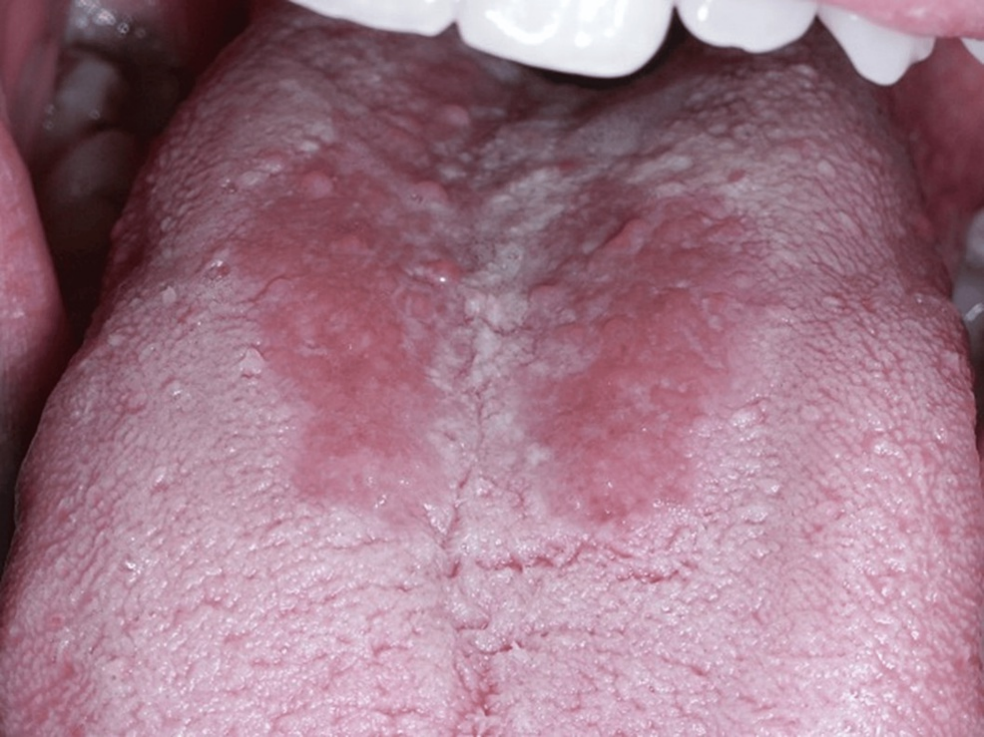
The oral manifestations included two circumscribed, erythematous atrophic mucosal areas with loss of papillae resembling geographic tongue, but without sufficient whitish regular borders.

A male patient, 24 years old, was referred to the private practice of one of the authors (DA), in February 2018, by the attending rheumatologist, with two painless, rounded lesions on the dorsal surface of the tongue. The patient reported that those two lesions persisted in the same location for more than six months. The patient, who never had skin psoriasis but had a family history of psoriasis (father), was clinically diagnosed with PsA and also fulfilled the CASPAR (ClASsification for Psoriatic ARthritis) criteria with Raynaud phenomenon. Clinically, in the oral cavity, two circumscribed, erythematous atrophic mucosal areas with loss of papillae resembling GT, but without sufficient whitish regular borders, were observed (Figure 1).

**Figure 2:**
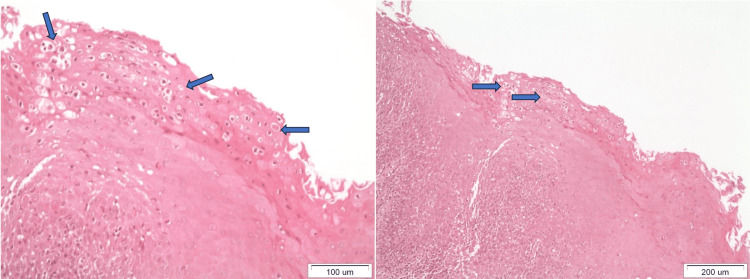
Histological examination. The blue arrows both in the left and the right panel show the presence of Munro’s microabscesses at the superficial epithelial layers, thus suggesting psoriasis-related tongue lesions similar to geographic tongue.

These lesions were persistent even after administration of anti-fungal (Fluconazole 150 mg) and antibacterial agents (Amoxicillin 500 mg and clavulanate 125 mg; Augmentin 625®). The biopsy that followed showed moderate hyperkeratosis, spongiosis, diffuse inflammatory infiltration of lymphocytes and neutrophils in the stratified squamous epithelium and the lamina propria, and Munro’s microabscesses at the superficial epithelial layers, thus suggesting psoriasis-related tongue lesions similar to GT (Figure 2).

The clinical appearance of oral lesions together with microscopic findings and the subsequent medical and family history suggested their relation to PsA. The patient is currently under treatment with secukinumab 300 mg (COSENTYX®), monthly, and methotrexate 20 mg (Metoject®), subcutaneously weekly, as his arthritis remains active.

## Discussion

Psoriasis is a prevalent inflammatory dermatological condition that impacts approximately 1-3% of the global population [23]. The occurrence of oral lesions in individuals with psoriasis is infrequent and subject to debate [17,22,24]. Several studies have indicated that the occurrence of oral lesions in psoriasis is either absent or infrequent [22]. The oral symptoms of psoriasis primarily encompass non-specific lesions, notably GT. Furthermore, it has also been postulated that these disorders exhibit an association with psoriasis [17,24]. GT is a prevalent oral lesion in individuals with psoriasis [16,19]. It exhibits clinical, histological, and genetic characteristics that closely resemble those of psoriasis, indicating that this oral lesion could potentially be considered an oral manifestation of psoriasis [17,24,25].

GT might potentially be considered an unsuccessful manifestation of psoriasis, which can progress with or without following co-occurrence [22]. The presence of a positive family history, a correlation between psoriasis and GT, and the existence of the histocompatibility antigen human leukocyte antigens (HLA)-Cw6 indicate a genetic foundation, so offering additional substantiation for the interconnectedness of these conditions. The correlation between psoriasis and genetic predisposition as well as familial history is supported by the observation that 35-42.4% of psoriasis patients have a positive family history [22]. Furthermore, the heightened prevalence of GT in individuals with severe psoriasis vulgaris indicates that this particular lesion could serve as an indicator of the severity of psoriasis [26-28].

The clinical characteristics of oral psoriasis exhibit significant variability and can manifest in any region of the oral mucosa, posing challenges in the diagnostic process [29]. Multiple oral lesions have been documented, exhibiting characteristics such as white or grey plaques, annular lesions, diffuse areas of erythema, and nonspecific lesions like geographic tongue [26].

GT occurrence rate ranges from 1% to 18% [24,26,30,31]. In particular, GT manifests a higher occurrence rate in individuals with psoriasis (12%) than in healthy individuals (3%) [22]. Another study concludes that 14% of the psoriatic patients experience GT [26]. A potential genetic link exists between psoriasis and GT [27]. The prognosis of psoriatic patients manifesting GT is worse than those who do not [22]. GT may be considered as a transient manifestation of the condition [22]. Early-onset psoriasis is seen as more severe and is linked to the presence of HLA-C*06, in contrast to late-onset psoriasis [32,33]. Individuals who develop psoriasis at or before the age of 30 can be classified as having early-onset psoriasis [33]. GT is more common in early-onset psoriatic patients than in the late-onset ones [24].

In our case, the familial history, the manifestation of GT, and the lack of typical psoriatic cutaneous lesions were considered as less favorable prognostic factors and the patient was advised to follow regular checkups so that any major exacerbation of the systematic symptoms could be preemptively avoided.

## Conclusions

Most of the oral manifestations in psoriasis are transient and only a minority of lesions can have a more permanent course. GT or relevant lingual lesions can be signs of psoriasis and can exist as a subclinical form of the condition. GT in early psoriasis is an indicator of disease severity and could be a preceding sign of future cutaneous involvement, thus potentially supporting an early diagnosis and monitoring of psoriasis/PsA.
